# Component resolved analysis of ash pollen allergy in Bavaria

**DOI:** 10.1186/s13223-018-0291-4

**Published:** 2018-11-08

**Authors:** Katharina Eder, Donata Gellrich, Catalina Meßmer, Martin Canis, Moritz Gröger

**Affiliations:** Department of Oto-Rhino-Laryngology, Head & Neck Surgery, Ludwig-Maximilians-University Munich, Marchioninistr. 15, 81377 Munich, Germany

**Keywords:** Ash pollen allergy, Native ash pollen extract diagnostic, rOle e 1, nOle e 7, rOle e 9

## Abstract

**Background:**

Sensitization to ash pollen is underestimated in various regions. The prevalence in Germany is about 10%. However, allergy to ash pollen is widely overlooked by allergists, since the pollination period of ash and birch in central Europe closely overlap and rhinoconjunctival symptoms during April/May are often assigned to birch pollen. Component resolved analysis of the different ash allergens is not routinely available. Therefore, we would like to question the usefulness of component resolved diagnostic via olive components, as ash and olive are both part of the Oleaceae family.

**Methods:**

113 patients with nasal provocation and skin prick test to ash were retrospectively compared regarding their specific immunoglobulin E antibody profiles with response to native ash extract, rOle e 1, nOle e 7 and rOle e 9.

**Results:**

In nasal provocation testing 58% of 113 patients sensitized to ash were allergic, 42% were only sensitized without showing symptoms. Skin prick testing and serology against native ash extract detected most patients sensitized to ash pollen, whereas rOle e 1 was less sensitive. However, the value of measurements of skin prick test, serology to native ash extract and rOle e 1 did not allow a differentiation between an allergy and clinically silent sensitization. Specific antibodies to nOle e 7 and rOle e 9 were only seen in individual patients and were all positive for native ash extract and rOle e 1.

**Conclusion:**

Skin prick testing and serology to native extract of ash pollen are the most reliable tools to diagnose a sensitization to ash pollen for patients living in Germany. Component resolved diagnostic to the major allergen rOle e 1 as representative of the Oleaceae family is possible but was less sensitive. Diagnostic of nOle e 7 and rOle e 9 did not show any additional benefit. Regarding differentiation between allergy and clinically silent sensitization to ash pollen, provocation is the leading diagnostic tool. Concluding, in routine clinical practice the standard methods—skin prick test, serology to native ash extract and provocation testing—remain crucial in the diagnosis and differentiation of ash sensitization and allergy.

## Background

Prevalences of allergic disease are increasing. Next to grass and herb pollen, also tree pollen are a major source of pollinosis, primarily birch-like plants. The prevalence of sensitization to birch pollen is 17.4% in the German adult population [1]. Therefore, this allergen accounts for various rhinoconjunctival symptoms during its season in April and May. However, sensitization to European ash pollen is widely underestimated in Germany as a reason for pollinosis during this time [2]. Since the pollination periods of ash and birch strongly overlap, sensitization to ash pollen should also be considered being the source of allergic symptoms during spring. Sensitization to ash pollen is determined as 9.4% in the German adult population [1]. In Italy and France, 18–34% of allergies are caused by ash pollen [2, 3].

The European ash tree (*Fraxinus excelsior*) is found in Germany and many other European countries except northern Scandinavia and the southern Mediterranean [4] and belongs to the Oleaceae family. Moreover, the olive tree (*Olea europaea*) is one of the major representatives of the Oleaceae family [5] and in Europe is mainly found in the Mediterranean regions and in regions of olive cultivation [4]. Alongside the major allergens Fra e 1 of the ash tree and Ole e 1 of the olive tree, there are a number of other allergens characterized for ash and olive tree, respectively. However, in contrast to recombinant olive components, none of the recombinant ash components are available for commercial testing in the UniCAP-FEIA (fluorescence enzyme immunoassay**)** system.

The major allergen Fra e 1 shows high cross-reactivity with the major allergen Ole e 1. Both major allergens are glycoproteins and share high sequential similarity [2, 6]. Therefore, Ole e 1 is considered a potent marker to diagnose sensitization to ash pollen [7, 8]. Next to Ole e 1, Ole e 7 and Ole e 9 are commercially available for testing in the UniCAP-FEIA system. Ole e 7 is a lipid transfer protein [9] and its prevalence varies dramatically between geographical areas of high and low pollen exposure [10–12]. Ole e 9 is a 1,3-ß-glucanase, the prevalence of this allergen is low, except in areas of extreme exposure to olive trees [13, 14]. Both allergens are believed to be linked to higher prevalence of asthmatic symptoms [10, 11]. However, the presence of specific immunoglobulin E (IgE) to ash or olive pollen and its components will strongly depend on the exposure to the allergen, which is varying within Europe and different regions.

In the European Union regulatory demands are rising for the production and use of any allergen solution used for diagnostic testing, favoring the use of “clean” component resolved analysis [15]. The standard tools, such as extract based SPT (skin prick test) and serology to native extracts, contain many of the allergenic molecules within one test and therefore, are very cost effective. On the other hand, these test solutions contain different components and partly at unknown concentrations, one reason for pushing component based diagnostic approaches.

Burbach et al. state a clinical relevance for sensitization to the Oleaceae family in Germany of about 50% [16]. Component based diagnostic tools might be useful to distinguish a clinically silent sensitized patient from an allergic patient. Several studies deal with this topic and point out that higher levels of specific IgE to e.g. Bet v 1, Der p 1 or Par j 2 could hint at a clinically relevant sensitization to birch, house dust mite or parietaria, but clinical relevance was not always proven by allergen challenge in these studies [17–19]. Especially in complex polysensitized patients the reported period of allergic symptoms during the year can only give an indication towards the causing allergen and provocation testing usually is needed to clearly identify the allergen causing these symptoms besides various sensitizations.

Therefore, we questioned if it is useful to follow the trend of component based diagnostic approach on the basis of olive tree components Ole e 1, Ole e 7 and Ole e 9 for the diagnosis of ash sensitization or allergy in Germany.

## Methods

### Patient data

The Department of Oto-Rhino-Laryngology, Head & Neck Surgery of the Ludwig-Maximilians-University in Munich runs an allergy data base that contains all patient information and diagnostic results of patients who visited the allergy department. We retrospectively scanned this database for patients who presented to our department between December 2009 and March 2017 and underwent nasal provocation testing (NPT) to ash pollen resulting in a total of 121 patients. We excluded patients who we could not clearly separate in ash allergic versus clinically silent sensitized, who did not receive SPT to ash at time of visit or if no serum was left for further analysis. 113 patients were left and included in this study. All patients were positively tested to ash in SPT and partly had received serum diagnostic to ash/olive allergy. In all patients diagnostic for specific IgE to native ash extract and the allergen components rOle e 1, nOle e 7 and rOle e 9 were completed.

### Nasal provocation test

In our study, beforehand all patients had received nasal provocation challenge with ash pollen to differentiate between allergy and sensitization without clinical relevance. The NPT was performed in accordance with the current guidelines [20]. The intranasal challenge test solution for ash was designated for nasal provocation and had an allergen concentration of 5.000 BE/ml (Allergopharma, Reinbek, Germany). Testing was considered positive if the intranasal airflow measured by rhinomanometry was decreased > 40% at 150 Pa on the side tested with the allergen, as well as for a symptom score > 3 or a decrease in intranasal airflow > 20% in combination with a symptom score > 2; the symptoms registered were secretion (0 = no secretion, 1 = little secretion, 2 = plenty secretion), irritation (0 = 0–2 × sneezing, 1 = 3–5 × sneezing, 2 = > 5 × sneezing) and remote symptoms (0 = no remote symptoms, 1 = lacrimation and/or itching of palate and/or itching of ears, 2 = conjunctivitis and/or chemosis and or urticaria and/or coughing and/or dyspnoe) [21].

### Skin prick test

The SPT solution for ash pollen by ALK-Abelló, Wedel, Germany was used. The SPT was considered positive with a wheal > 3 mm in diameter (I = ≥ 3 to 4, II = ≥ 4 to 5, III = ≥ 5 to 6, IV = ≥ 6) in combination with Histamine dihydrochloride solution at 1 mg/ml as positive control and allergen-free saline solution as negative control. It was read 20 min after application. The procedure and classification were in line with European standards and published guidelines [22–26].

### Fluorescence enzyme immunoassay (FEIA)

The FEIA method (UniCAP-FEIA, Thermo Fisher Scientific, Freiburg, Germany) was used to detect IgE reactivity to purified native ash allergen extract and allergen components rOle e 1, nOle e 7 and rOle e 9 with a commercially available test kit (Thermo Fisher Scientific, Freiburg, Germany). All procedures were in accordance with the manufacturer´s instructions. The results are given as ratio of specific IgE in concentration units (kU/l) and total IgE in concentration units (kU/l). The positive cutoff value was > 0.35 kU/l as suggested by the manufacturer.

### Statistical analyses

Statistical analysis was performed on a Lenovo Thinkpad X61 s with SigmaPlot (Jandel Corp., San Rafael, CA, USA) and Excel (Microsoft, Redmond, WA, USA). The median was used for descriptive statistics since all data failed normality testing (Shapiro–Wilk). The Mann–Whitney Rank Sum Test was utilized for testing statistically significant differences in the median values between the two groups. Correlation between native ash extract and rOle e 1 results was calculated by Spearman Rank Order Correlation with absolute values of specific IgE in kU/l. A *p*-*value *<0.05 was considered significant.

## Results

In our study, a total of 113 patients had received NPT with ash pollen. Based on this we divided the patient collective in 2 groups: ash pollen allergy and sensitization to ash without clinical relevance. 66 (58%) patients had an allergy to ash pollen, 47 (42%) patients showed a sensitization to ash without eliciting clinical symptoms. Table 1 summarizes the patient data.

**Table 1:** Demographics and characteristics of patients with ash NPT

	Silent sensitization (n = 47/42%)	Allergy (n = 66/58%)
Male	34 (72%)	40 (61%)
Female	13 (28%)	26 (39%)
Age (range 5–76 years)	33.4	32.7
Mono-sensitized	1 (2%)	2 (3%)
Oligo-sensitized	2 (4%)	6 (9%)
Poly-sensitized	44 (94%)	58 (88%)
Co-sensitization to		
Other trees	45 (96%)	58 (88%)
Herbs	21 (45%)	28 (42%)
Grass	30 (64%)	42 (64%)
Animals	30 (64%)	38 (56%)
Mold	11 (23%)	16 (24%)
Mite	23 (49%)	35 (53%)
Latex	2 (4%)	1 (2%)
Asthma	21 (45%)	32 (49%)
Food allergy	13 (28%)	27 (41%)

Values are number of patients total and percent of each evaluated group

Age is given as a median

In terms of gender distribution, age, sensitization profiles and reported asthma both groups were comparable. We distinguished between patients mono-sensitized to ash, patients that were oligo-sensitized to 1–2 additional perennial or seasonal allergens, and patients poly-sensitized to 3 or more allergens in addition to ash pollen. In summary, we looked at sensitizations to other trees, herbs, grass, animal dander, mold, mite and latex. Most patients, 94% of sensitized patients and 88% of allergic patients, were poly-sensitized. Also, 97 patients (86% of all patients) had a positive result in SPT with birch tree. In addition, an equivalent percentage of patients in the allergy and in the sensitization group reported asthma, 45% of patients in the sensitization group and 49% in the allergy group. In terms of reported food allergy, we found a slightly higher percentage of patients in the allergy groups namely 41% compared to 28% in the sensitization group.

We compared different diagnostic tools within the allergy and sensitization groups (Table 2). All patients had a positive SPT for ash pollen. The median values of the SPT for ash pollen were III (range II–IV) in the sensitization group and IV (range I–IV) in the allergy group. However, this difference was not statistically significant (*p *=* 0.714*). In terms of sensitization profiles of allergic and asymptomatic patients we compared native ash extract and the olive tree components rOle e 1, nOle e 7 and rOle e 9, as representative components of the Oleaceae family.

**Table 2:** SPT and sensitization profile to native ash extract and different olive components

	Silent sensitization (n = 47/42%)	Allergy (n = 66/58%)	*p*-*value*
SPT	III (II–IV)	IV (I–IV)	*0.714*
Native ash positive	46 (98%)	65 (99%)	
IgE level/total IgE level	0.015 (0.2–60.1)	0.012 (0.25–100)	*0.176*
rOle e 1 positive	41 (87%)	58 (88%)	
IgE level/total IgE level	0.010 (0–66.3)	0.007 (0–100)	*0.197*
nOle e 7 positive	1 (2%)	2 (3%)	
IgE level/total IgE level	< 0.001 (0–0.75)	< 0.001 (0–0.77)	No *p*
rOle e 9 positive	0 (0%)	2 (3%)	
IgE level/total IgE level	< 0.001 (0–0.32)	< 0.001 (0–2.14)	No *p*

SPT results are given as median and range

Values of serum diagnostic approaches are number of patients in total and percent of each evaluated group

IgE level ratios are given as median (range is shown as total values in kU/l)

**Fig. 1:**
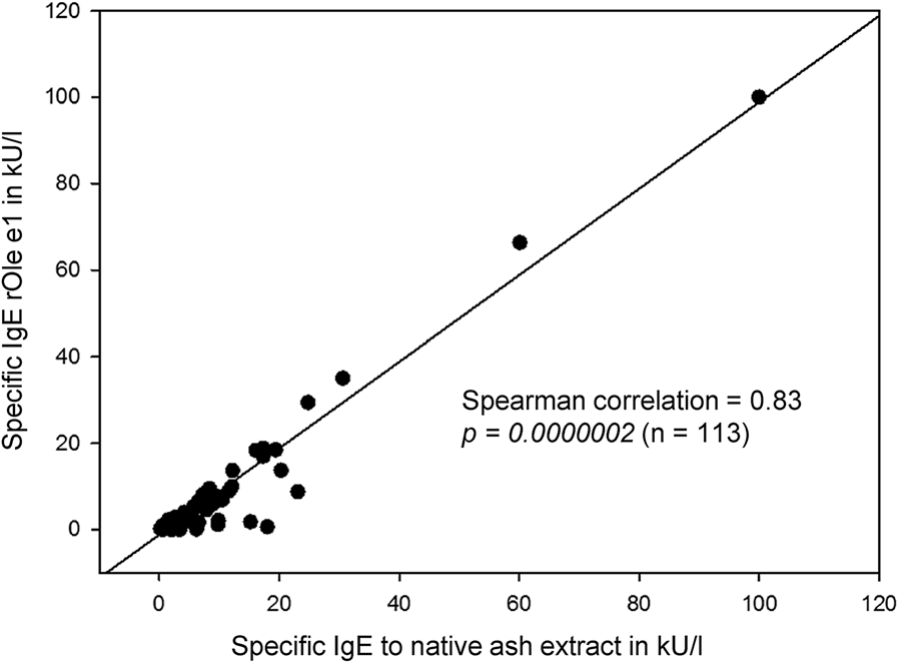
Spearman Correlation of specific IgE to rOle e 1 and to native ash extract (n = 113). Levels of specific IgE to rOle e 1 and to native ash extract highly correlate with a correlation coefficient of 0.83 (*p *=0.0000002)

Prevalence of a specific IgE to native ash extract was 99% in the allergy group and 98% in the sensitization group. Results are shown as ratio to total IgE, median values were 0.012 and 0.015, respectively. The two patients who were negative for the native extract according to the manufacturer´s suggested positive cutoff value of > 0.35 kU/l, in fact showed values of specific IgE to native extract of 0.2 and 0.25 kU/l. Both had a positive SPT (II and IV) and both showed low values for specific IgE to rOle e 1 (0.18 and 0.2 kU/l). Antibodies to the major allergen rOle e 1 were present in 88% of patients in the allergy group and in 87% of patients in the sensitization group. The median value of antibodies to rOle e 1 in relation to total IgE was 0.007 in the allergy group and 0.010 in the sensitization group. Median values of antibodies to native ash extract and rOle e 1 did not differ significantly between both groups (*p *=* 0.176* for native ash extract and *p *=* 0.197* for rOle e 1). However, specific IgE to native ash extract and rOle e 1 highly correlated with a correlation coefficient of 0.83 (Spearman correlation *p *= *0.0000002)* (Fig. 1). Prevalences of nOle e 7 and rOle e 9 were very low in both groups. In the allergy group only two patients had specific antibodies to the allergens nOle e 7 (3%) and two patients to rOle e 9 (3%), in the sensitization group it was only one patient with nOle e 7 (1%), no patient had specific IgE to rOle e 9. Patients having specific antibodies to nOle e 7 or rOle e 9 also had specific IgE to native ash extract and rOle e 1. All of these 5 patients reported asthma, but only two reported a food related allergy.

## Discussion

This study investigated native ash extract-based diagnostic approaches and the use of component based diagnostic tools in patients sensitized to European ash pollen in routine clinical practice in Germany, since ash and olive tree belong to the Oleaceae family and cross-reactivity between the two has been widely seen.

Our study collective does not represent the main population. The majority of patients are poly-sensitized indifferent of the existence of symptoms. This is due to the inclusion criterium of nasal provocation with ash pollen to clearly separate between clinically silent sensitizations and allergy. Especially complex poly-sensitized patients are in need of provocation testing to relate symptoms to a specific sensitization. In mono- or oligo-sensitized patients, the reported time of the year, the patient is suffering from symptoms, usually is in line with an individual sensitization. Therefore, mono-sensitized and oligo-sensitized patients are most likely underrepresented in this study, which leads to certain limitations that will be mentioned in the course of the discussion. On the other hand, especially complex poly-sensitized patients should benefit from component based diagnostic approaches.

Allergy to ash is frequently underestimated in Germany [2]. An elaborate diagnostic approach is required to conclude possible therapeutic strategies in patients with clinical symptoms during March until May since ash and birch tree strongly overlap in their pollination period. In our patient collective 86% also had a positive SPT with birch. Testing ash sensitization via specific IgE to native extract is almost as effective as SPT to ash, 98% of our patients showed specific IgE. On the other hand, we would have missed two patients having levels below the positive cutoff value by testing specific IgE to native ash extract alone. Component based diagnostic of sensitization to ash via testing for specific IgE to Ole e 1 is described as comparably effective in the literature [7], which could not be seen in our study. The above mentioned two patients without IgE to native extract also showed low levels of specific IgE to Ole e 1 below the positive cutoff value. Furthermore, twelve additional patients did not have specific IgE to Ole e 1 above the positive cutoff value of > 0.35 kU/l and would have been overlooked with an exclusive component based diagnostic approach. 9 of these 14 missed sensitizations were clinically relevant. Concluding, based on our study results native extract approaches such as SPT and specific IgE to native extract remain crucial in the diagnosis of sensitization and allergy in routine clinical practice and cannot been replaced by component based diagnostic testing via rOle e 1 alone.

Presence of antibodies to nOle e 7 and rOle e 9 was rare in our patient collective. Only 5 patients in total showed positive values to one of these components. It is known that the prevalence of olive components depends on geographical location [27]. Sensitization to nOle e 7 and rOle e 9 strongly depends on the intensity of exposure to olive pollen [12–14]. Therefore, sensitization via ash pollen did not result in a specific immune response that could be detected by nOle e 7 and rOle e 9 serology in our study collective. Concluding, these components are not suitable for general diagnosis of sensitization and allergy to the ash tree in Germany, but of course may be crucial in other European countries, when sensitization occurs via olive tree.

It is reported for sensitization to e.g. cat that a high value in SPT and native extract serology can indicate the presence of an allergy versus a clinically silent sensitization showing lower values [28]. This could not be seen for ash allergy and/or sensitization. Differences in SPT and serology of native ash extract or rOle e 1 were not statistically significant for both groups. Nevertheless, this might be due to the patient collective as mentioned above. Mono- and oligo-sensitized patients are underrepresented which could alter the results accordingly. Therefore, in complex poly-sensitized patients, NPT with ash pollen remains crucial in the diagnosis of a manifest allergy.

Sensitization to nOle e 7 and rOle e 9 is documented to be associated with asthma [10, 14]. Indeed, all patients with specific antibodies to the one or other allergen reported asthma. On the other hand, all nOle e 7 or rOle e 9 sensitized patients were poly-sensitized and in total 53 patients (47% of all patients) reported asthma in this study. Both groups, patients only sensitized and patients allergic to ash, show an equal percentage of polysensitization as well as asthma. Following, the majority of patients reporting asthma did not show any sensitization to nOle e 7 or rOle e 9. Therefore, we conclude that asthma in these patients is not a specific nOle e 7 or rOle e 9 effect.

Also, it was shown, that the presence of specific IgE to Ole e 7 is associated with higher risk for food induced anaphylaxis [14, 29]. Only 3 patients in our study had specific IgE to Ole e 7 and only one of them reported oral symptoms after the consumption of certain fruit but no severe anaphylactic reaction. Ole e 9 is presumed to be involved in the pollen-Latex-fruit-syndrome [14, 30]. Two patients in our study had specific IgE to Ole e 9, but none of them had a positive SPT for Latex or reported symptoms associated with the pollen-Latex-fruit-syndrome. However, the low prevalence of Ole e 7 and Ole e 9 in our study should not lead to any conclusion regarding food related symptoms.

In conclusion, this study provides evidence that testing for the olive components rOle e 1, nOle e 7 and rOle e 9 alone is not sufficient to reliably identify ash sensitized and ash allergic patients. In routine clinical practice diagnosing an allergy to European ash in Germany should be based on extract-based methods, such as the SPT and serology to native ash extract. Replacing these standard tools by only recombinant diagnostic with rOle e 1 or other olive allergen components could miss sensitized patients. Moreover, differentiation between manifest allergy to ash and clinically silent sensitization can only be reliably performed via NPT at least in poly-sensitized patients. In routine clinical practice in Germany, determination of serology for the commercially available olive components nOle e 7 and rOle e 9 does not seem to deliver any additional benefit in the diagnosis of ash allergy.

## Abbreviations

FEIA: fluorescence enzyme immunoassay
SPT: skin prick test
IgE: immunoglobulin E
NPT: nasal provocation testing
